# Supplementary material for: Transcriptional Regulation of Siglec-15 by ETS-1 and ETS-2 in Hepatocellular Carcinoma Cells
Source: Int J Mol Sci. 2023 Jan 2;24(1):792. doi: 10.3390/ijms24010792 (PMC9821606; doi:10.3390/ijms24010792)
Supplement: Supplementary file 1 [file ijms-24-00792-s001.zip › ijms-2020716-supplementary.pdf]

Table S1. The sequences of primers used for PCR amplification, qRT-PCR and EMSA

primer	sequence
-1800nt~+55nt	F: cctgagctcgctagcctcgagTATGGGATGCCAAGTGGCG
-1540nt~+55nt	F: cctgagctcgctagcctcgagGACAGTAGACCAGCCTCTGGGC
-1270nt~+55nt	F: cctgagctcgctagcctcgagACCGTGTTAGCCAAGATGGTCT
-942nt~+55nt	F: cctgagctcgctagcctcgagTAGCTCAGCTTTCCTAGAAGGATAATG
-735nt~+55nt	F: cctgagctcgctagcctcgagCCAAGTGCCAAGGGAAGGG
-453nt~+55nt	F: cctgagctcgctagcctcgagGAGAGGGAGGGAGGAATAAGGG
-231nt~+55nt	F: cctgagctcgctagcctcgagTCTTTCTCACCCTCTTTCCTGTTT
-21nt~+55nt	F: cctgagctcgctagcctcgagCCACGCCTGGGAGGGCCC
-1800nt~+55nt	R: cagtaccggattgccaagcttTGCTGTGAGCATCTGAACACCC
pcDNA3.1-ETS-1-Flag:	F: gggagacccaagctggctagc ATGAAGGCGGCCGTCGATCTCAA
	R: tccgagctcggtaccaagcttTCACTTGTCGTCATCGTCTTTGTAGT CCTCGTCGGCATCTGGCTTG
pcDNA3.1-ETS-2-Flag:	F: gggagacccaagctggctagcATGAATGATTTCGGAATCAAGAATA TGGACC
	R: tccgagctcggtaccaagcttTCACTTGTCGTCATCGTCTTTGTAGT CGTCCTCCGTGTCGGGC
Siglec-15- qRT-PCR	F: TTTGAGCCAGATGAACCC CC
	R: CAGGGAGCTCCGAAATG GTT
GAPDH- qRT-PCR	F: GGTGTGAACCATGAGAAGTATGA
	R: GAGTCCTTCCACGATACCAAAG
ETS-1- qRT-PCR	F: ACGTGTACCGCTTTGTGTGT
	R: TCGTCGGCATCTGGCTTG
ETS-2- qRT-PCR	F: CTCTGGGCCACCAATGAGTT
	R: TCACCCACAAAGTCAGGTGC
Cold EBS1 probe	F: GGAGGGAGAAAGGGAGGAAGAGAGG
	R: CCTCTCTTCC TCCCTTTCTC CCTCC
Biotin EBS1 probe	F: GGAGGGAGAAAGGGAGGAAGAGAGG
	R: CCTCTCTTCC TCCCTTTCTC CCTCC
Mutated EBS1 probe	F: GGAGGGAGAAAGAAAAAAAGAGAGG
	R: CCTCTCTTTT TTTCTTTCTC CCTCC
Cold EBS2 probe	F: AGGAAGAGAGGAAGGAAGGAA
	R: TTCCTTCCTT CCTCTCTTCCT
Biotin EBS2 probe	F: AGGAAGAGAGGAAGGAAGGAA
	R: TTCCTTCCTT CCTCTCTTCC T
Mutated EBS2 probe	F: AGGAAGAAAAAAAAAAGGAA

	R: TTCCTTTTTT TTTTTCTTCC T
Cold EBS3 probe	F: AAGGAAAGGAAGGAGGGAGGGAGA
	R: TCTCCCTCCC TCCTTCCTTT CCTT
Biotin EBS3 probe	F: AAGGAAAGGAAGGAGGGAGGGAGA
	R: TCTCCCTCCC TCCTTCCTTT CCTT
Mutated EBS3 probe	F: AAAAAAAAAAAGAGGGAGGGAGA
	R: TCTCCCTCCC TCTTTTTTTT TTTT
Cold EBS4 probe	F: GAGGGAGGGAGAGGAAGGAAGTGAGGGAGGGA
	R: TCCCTCCCTC ACTTCCTTCC TCTCCCTCCC TC
Biotin EBS4 probe	F: GAGGGAGGGAGAGGAAGGAAGTGAGGGAGGGA
	R: TCCCTCCCTC ACTTCCTTCC TCTCCCTCCC TC
Mutated EBS4 probe	F: GAGGGAGGGAGAAAAAAAAAGTGAGGGAGGGA
	R: TCCCTCCCTCACTTTTTTTTTCTCCCTCCCTC
Cold EBS5 probe	F: GAGGGAGGGAGGAAGGAAGGGTGAGCCACAT
	R: ATGTGGCTCA CCCTTCCTTC CTCCCTCCCT C
Biotin EBS5 probe	F: GAGGGAGGGAGGAAGGAAGGGTGAGCCACAT
	R: ATGTGGCTCA CCCTTCCTTC CTCCCTCCCT C
Mutated EBS5 probe	F: GAGGGAGGGAGGAAAAAAAGGTGAGCCACAT
	R: ATGTGGCTCA CCTTTTTTTC CTCCCTCCCT C
Cold EBS6 probe	F: GATGGCAGCCACAGGAAATGGCCCTGTT
	R: AACAGGGCCA TTTCCTGTGG CTGCCATC
Biotin EBS6 probe	F: GATGGCAGCCACAGGAAATGGCCCTGTT
	R: AACAGGGCCA TTTCCTGTGG CTGCCATC
Mutated EBS6 probe	F: GATGGCAGCCAGAAAAAATGGCCCTGTT
	R: AACAGGGCCA TTTTTTCTGG CTGCCATC
TGF-β1-Cold EBS-1-2 probe	F: CTTTCTCACCCTCTTTCCTGTTTCCTGTCCTGGA
	R: TCCAGGACAGGAAACAGGAA AGAGGGTGAG AAAG
TGF-β1-BiotinEBS-1-2probe	F: CTTTCTCACCCTCTTTCCTGTTTCCTGTCCTGGA
	R: TCCAGGACAGGAAACAGGAA AGAGGGTGAG AAAG
TGF-β1-MutatedEBS-1-2probe	F: CTTTCTCACCCTCTAAGGTATTTAAGGTCCTGGA
	R: TCCAGGACCT TAAATACCTT AGAGGGTGAG AAAG
TGF-β1-Cold EBS3 probe	F: GGCTGCCAGGCTTCCTGTCCCCAGTTC
	R: GAACTGGGGACAGGAAGCCTGGCAGCC
TGF-β1-Biotin EBS3 probe	F: GGCTGCCAGGCTTCCTGTCCCCAGTTC
	R: GAACTGGGGATACCAACCCTGGCAGCC
TGF-β1-Mutated EBS3 probe	F: GGCTGCCAGGGTTGGTATCCCCAGTTC
	R: GAACTGGGGACAGGAAGCCTGGCAGCC
TGF-β1-Cold EBS4 probe	F: CCTGTCCCCAGTTCCTCCGGCTCCCGCAGA
	R: TCTGCGGGAGCCGGAGGAACTGGGGACAGG

TGF-β1-Biotin EBS4 probe	F: CCTGTCCCCAGTTCCTCCGGCTCCCGCAGA
	R: TCTGCGGGAGCCGGAGGAACTGGGGACAGG
TGF-β1-Mutated EBS4 probe	F: CCTGTCCCCAGTTTATCCGGCTCCCGCAGA
	R: TCTGCGGGAGCCGGATAAACTGGGGACAGG
TGF-β1-Cold EBS5 probe	F: CCGCCCGCCTTCCTTCCCCCACCACGC
	R: GCGTGGTGGGGGAAGGAAGGCGGGCGG
TGF-β1-Biotin EBS5 probe	F: CCGCCCGCCTTCCTTCCCCCACCACGC
	R: GCGTGGTGGGGGAAGGAAGGCGGGCGG
TGF-β1-Mutated EBS5 probe	F: CCGCCCGCGAAGGTTCCCCCACCACGC
	R: GCGTGGTGGGGGAACCTTCGCGGGCGG

Table S2. The sequences of siRNAs used for qRT-PCR, Western blot and Dual-luciferase reporter assay

siRNA	sequence
ETS-1-2	F: GCACCUUCAAGG-ACUAUGUTT
	R: ACAUAGUCCUUGAAGGUGCTT
ETS-1-3	F: GCAGUUUCUUCUGGAAUUATT
	R: UAAUUCCAGAAGAAACUGCTT
ETS-2-1	F: AGAAAUGGAAUCCAAGCCUGUUGG
	R: CCAACAGGCUUGGAUUCCAUUUCU
ETS-2-2	F: GCCAACAGGCUUGGAUUCCAUUUCU
	R: AGAAAUGGAAUCCAAGCCUGUUGGC

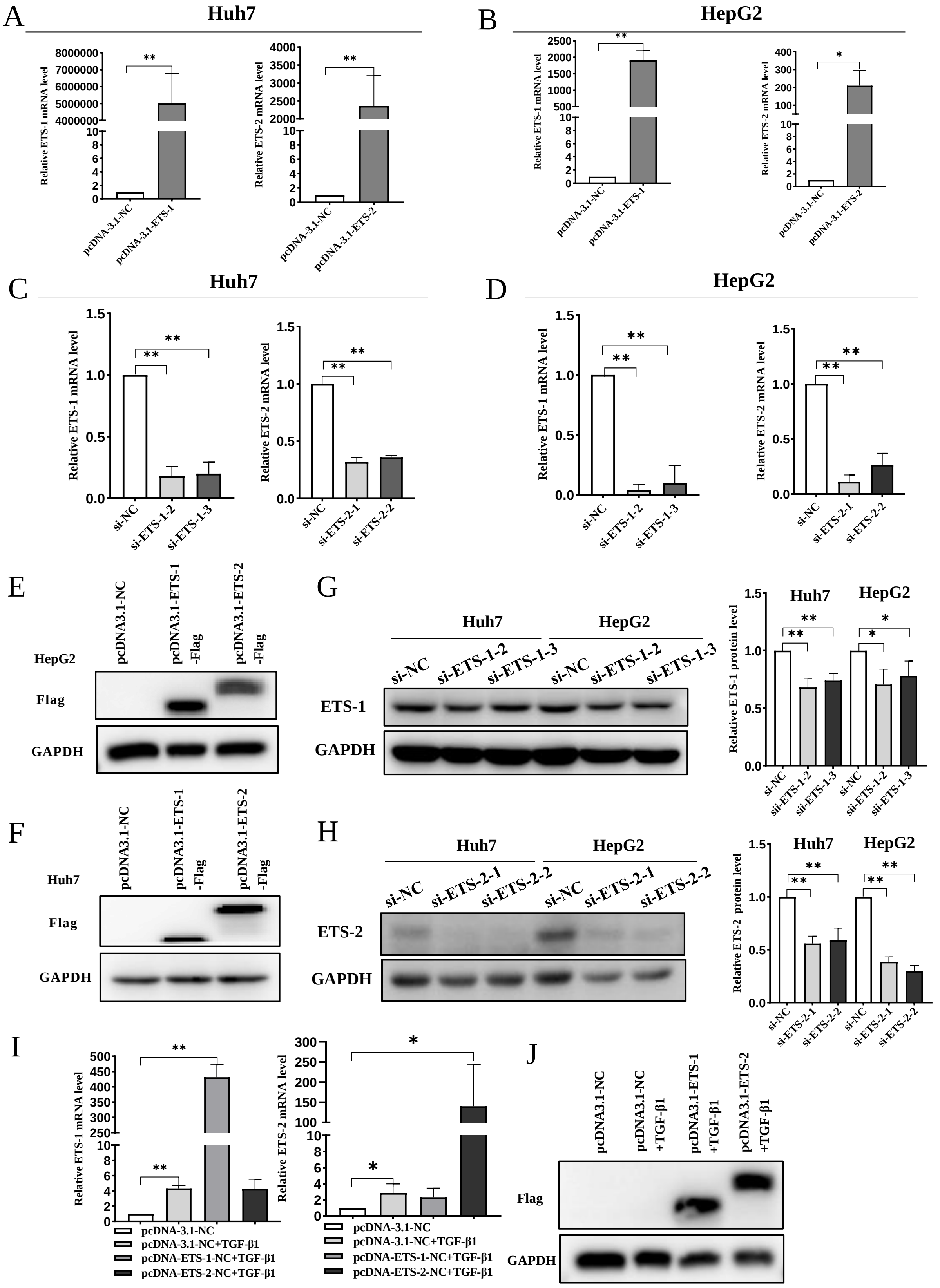

Supplementary Figure S1

Figure S1. (A-H) The specificity and efficiency of ETS-1 and ETS-2 overexpression or knockdown at mRNA (A-D) and protein (E-H) levels was confirmed in HepG2 and Huh7 cells by qRT-PCR and Western blot analysis, respectively. (I-J) The specificity and efficiency of ETS-1 and ETS-2 overexpression at mRNA (I) and protein (J) levels was confirmed in HepG2 cells by qRT-PCR and Western blot analysis, respectively. * $p < 0.05$ and ** $p < 0.01$.
